# Linking Coping Behavior and Mental Well-Being in Older Adults During the COVID-19 Pandemic

**DOI:** 10.1093/geroni/igab046.2680

**Published:** 2021-12-17

**Authors:** Dugan O'Connor, Jennifer Smith

**Affiliations:** Mather, Evanston, Illinois, United States

## Abstract

In addition to being a significant source of stress, the COVID-19 pandemic required older adults to drastically alter their behaviors and routines. People cope with stress in various ways; however, the pandemic was a unique situation that warranted investigation of this topic. This study aimed to identify the ways older adults coped with the COVID-19 pandemic, and the relationship between specific coping behaviors and perceived stress and mental health. Two hundred thirty-one older adults, ages 53 to 90, completed an online survey about coping behaviors used to manage stress during the pandemic, as well as measures of loneliness, depression, perceived stress, and the negative impact of the COVID-19 pandemic on their lives. “Talking with friends and family” (83%) and “increased television watching or other screen-time” (68%) were the most common coping behaviors. A series of one-way analyses of covariance (ANCOVA), with race, gender, age, education, and income included as covariates, revealed “eating more often” and “drinking alcohol” were associated with greater loneliness, depression, and stress. “Increased screen time” was also associated with greater depression and stress. “Engaging in more family activities,” was associated with a less negative impact of the pandemic, and “talking with friends and family” was associated with less loneliness. These findings suggest older adults who coped with stress of the COVID-19 pandemic through more eating, drinking, and the second most common behavior—watching TV—were more likely to report poorer well-being, and may benefit from programs to boost virtual social engagement.

